# Dauricine Protects from LPS-Induced Bone Loss via the ROS/PP2A/NF-κB Axis in Osteoclasts

**DOI:** 10.3390/antiox9070588

**Published:** 2020-07-06

**Authors:** Hyun-Jung Park, Malihatosadat Gholam Zadeh, Jae-Hee Suh, Hye-Seon Choi

**Affiliations:** 1 Department of Biological Sciences, University of Ulsan, Ulsan 44610, Korea; oli_jjung@naver.com (H.-J.P.); gholamzadeh.malihe@gmail.com (M.G.Z.); 2 Department of Pathology, Ulsan University Hospital, Ulsan 44030, Korea; drjhsuh1@gmail.com

**Keywords:** dauricine, inflammatory bone loss, osteoclast, PP2A, ROS

## Abstract

Dauricine (DAC), an isoquinoline alkaloid, exhibits anti-inflammatory activity. We hypothesized that DAC may prevent the inflammatory bone loss induced by lipopolysaccharide (LPS). LPS-induced bone loss was decreased by DAC in female C57BL/6J mice as evaluated by micro-computerized tomography (μCT) analysis. In vivo tartrate-resistant acid phosphatase (TRAP) staining showed that the increased number of osteoclasts (OCs) in LPS-treated mice was attenuated by DAC, indicating that DAC exhibited bone sparing effects through acting on OCs. DAC also decreased the differentiation and activity of OCs after LPS stimulation in vitro. LPS-induced cytosolic reactive oxygen species (cROS) oxidized PP2A, a serine/threonine phosphatase, leading to the activation of IKKα/β, followed by the nuclear localization of p65. DAC decreased LPS-induced ROS, resulting in the recovery of the activity of PP2A by reducing its oxidized form. Consequently, DAC reduced the phosphorylation of IKKα/β to block the nuclear localization of p65, which decreased NF-κB activation. Taken together, DAC reduced the differentiation and activity of OCs by decreasing ROS via the ROS/PP2A/NF-κB axis, resulting in protection from LPS-induced bone loss. We have demonstrated that LPS-induced bone loss was inhibited by DAC via its action on OCs, implying the therapeutic potential of DAC against inflammatory bone loss.

## 1. Introduction

Inflammatory diseases such as rheumatoid arthritis, psoriatic arthritis, and Crohn’s disease have been reported to be associated with severe bone loss [1,2,3]. Inflammation-induced bone loss affects the stability of the skeleton, leading to an increased fracture risk [4]. Although immune cells, mesenchymal cells, and the neighboring microenvironment all affect inflammatory bone destruction, osteoclasts (OCs) are key players in osteolysis [5]. Immune cells and their related products affect bone density through their effects on bone cells. Activated monocytes have been demonstrated to produce excess IL-1β that directly promotes bone resorption [6]. In addition, receptor activator of nuclear factor kappa-B ligand (RANKL), tumor necrosis factor-α (TNF-α), and IL-17 contribute to bone loss via influencing bone cells [7]. The secreted products of immune cells infiltrating due to inflammation maintain the inflammatory response, which initially recruits OC and then enhances OC formation and OC activity. In animal models, lipopolysaccharide (LPS) treatment induces systemic inflammation along with severe bone loss via the activity of OCs [8,9,10]. In vitro, LPS has been reported to enhance the differentiation, survival, and function of OCs [11,12,13,14].

OCs are derived from hematopoietic cells, and the OC precursors undergo differentiation and then further processes to become bone-resorbing cells. A critical role of NF-κB signaling in OC differentiation has been confirmed by severe osteopetrosis in NF-κB p50/p52 double-knockout mice with defective OC differentiation [15]. In the resting state, NF-κB dimers form complexes with inhibitory IκB proteins that block the nuclear localization signal of the dimers and inhibit their nuclear localization and binding to DNA, preventing their activation [16].

Classical NF-κB signaling is initiated upon the binding of ligands to their corresponding receptors—including TNFR, RANK, and IL-1R—which activate an IKK complex containing IKKα, IKKβ, and IKKγ. The activation of IKK triggers the degradation of IκB, which in turn frees the NF-κB dimers and facilitates their nuclear translocation, leading to the DNA binding of NF-κB p50:p65 heterodimers [16]. The inactivation of IKK’s catalytic activity protects against newly synthesized IκB proteins. Conditional IKKβ deficiency leads to osteopetrosis with a reduced number of OCs, whereas IKKα is not necessary for osteoclastogenesis in vivo, suggesting a critical role of IKKβ as a signal transducer for NF-κB dimers in OC differentiation [17].

Dauricine (DAC) is an isoquinoline alkaloid derived from a root used in traditional Chinese medicine, *Rhizoma menispermi*. Its pharmacological efficacy has been demonstrated in various inflammatory diseases [18,19,20,21]. DAC protects against the inflammation induced by ischemia/reperfusion in rats with reduced mRNA levels of IL-1β and TNF-α [18]. Inflammation-induced lung injury has been ameliorated by the administration of DAC, which suppressed the LPS-induced nuclear translocation and the transcriptional activity of NF-κB by attenuating the phosphorylation of IκB in vitro [19]. DAC also induces apoptosis in colon cancer cells via suppressing NF-κB signaling [20]. The neuroprotective effects of DAC have been demonstrated in *Caenorhabditis elegans* GMC101, an Alzheimer’s disease model system, with reduced oxidative damage through the activation of Nrf2 [21]. The antioxidant activity of DAC has also been reported in ethanol extracts of lotus, which contains various alkaloids including DAC [22].

Our previous studies have demonstrated a protective role of NF-κB signaling blockade against inflammatory bone loss [9]. That prompted us to hypothesize that DAC might prevent inflammation-induced bone loss by acting as an inhibitor of NF-κB signaling. We investigated the detailed molecular mechanisms of how DAC blocks LPS-induced bone loss via acting on OCs.

## 2. Materials and Methods

### 2.1. Animals and Study Design

Ten-week-old C57BL/6J female mice were maintained in the specific pathogen-free (SPF) facility of the Immunomodulation Research Center (IRC), University of Ulsan. All the mice were randomly split into four groups (*n* = 5 per group). Each group was injected intraperitoneally (i.p.) for 3 weeks with the (1) vehicle control (PBS); (2) vehicle with dauricine (DAC, Chengdu Biopurity Phytochemicals Ltd. Chengdu, Sichuan, China) (2.5 mg/kg, freshly prepared in PBS with 1 N HCl and adjusted to pH 7.2 with NaOH, once every two days); (3) LPS (Sigma–Aldrich, St. Louis, MO, USA) (5 mg/kg, once a week) [23]; and (4) LPS with DAC. All procedures were carried out according to the guidelines of the Institutional Animal Care and Use Committee (IACUC) of the IRC. The approval ID is #HSC-16-010. Blood samples were collected retro-orbitally under anesthesia prior to sacrifice. The right femur was evaluated using a high-resolution micro CT (μCT) SkyScan 1176 System (Bruker-Micro CT, Kontich, Belgium) for the analysis of the bone mineral density (BMD) and microarchitecture, following the methods of Park et al. [9]. The serum concentrations of collagen-type I fragment (CTX-1) (with the RatLaps EIA assay kit, Immunodiagnostic Systems Inc., Fountain Hills, AZ, USA), osteocalcin (with the osteocalcin EIA kit, Biomedical Technologies Inc., Stoughton, MA, USA), alkaline phosphatase (ALP) (with the ALP colorimetric determination kit, BioAssay Systems, Hayward, CA, USA), reactive oxygen species (ROS) (with OxiSelectTM hydrogen peroxide, Cell Biolabs Inc., San Diego, CA, USA), and MCP-1 (with a kit from R & D Systems, Inc., Minneapolis, MN, USA) were assessed according to each manufacturer’s protocol.

### 2.2. OC Formation

Whole bone marrow cells were harvested from 4–5-week-old C57BL/6J mice as described in [24], and further steps were performed according to the methods of Park et al. [9]. After fixation and staining, the number of tartrate-resistant acid phosphatase (TRAP)-positive multinucleated cells (MNCs) (with three or more nuclei), area, maximum diameter, and fusion index were evaluated as previously described [9].

### 2.3. Cell Viability

Equal numbers of bone marrow derived macrophages (BMMs) as prepared above were seeded to determine cell viability by the MTT assay as described [24].

### 2.4. RNA Isolation and Quantitative Polymerase Chain Reaction (qPCR)

Total RNA was isolated using QIAzol reagent as described by Park et al. [9]. Relative gene expression was calculated using the formula 2^−∆∆Ct^ with normalization to RPS. The primer sequences were used as described [9].

### 2.5. Bone Resorption

To evaluate the bone resorption activity of OCs, dentine slices were used to incubate mature OCs [25]. Pre-osteoclasts were cultured with M-CSF (R & D Systems, Inc.) and RANKL (R & D Systems, Inc.) to obtain mature OCs for 3–4 days. Mature OCs were released from the dishes, and seeded on top of the dentine slices for further incubation with M-CSF (30 ng/mL) and LPS (50 ng/mL) in the presence or absence of DAC (7 μM) for 4 days. The dentine slices were stained and evaluated by the methods of Park et al. [9].

### 2.6. Western Blot Analysis

Protein extraction, separation by SDS-PAGE, transfer to nitrocellulose membranes, blocking, incubation with secondary antibodies, and development were performed as previously described [10]. Primary antibodies against phospho-IKKα/β (S176/180) (#2697, Cell Signaling, Danvers, MA, USA), lamin B (#13435, Cell Signaling), p65 (sc-372, Santa Cruz Biotechnology, Santa Cruz, CA, USA), PP2A (sc-13600, Santa Cruz Biotechnology), and β-actin (A5441, Sigma–Aldrich) were incubated with the membranes overnight at 4 °C.

### 2.7. Determination of Intracellular and Mitochondrial Reactive Oxygen Species (ROS)

The fluorescent probe 2’,7’-dichlorofluorescein diacetate (H_2_DCFDA) (Thermo Fisher Scientific, Waltham, MA, USA) and the Mito-SOX red mitochondrial superoxide indicator (Invitrogen, Carlsbad, CA, USA) were used to evaluate intracellular ROS and mitochondrial ROS, respectively, as described [10].

### 2.8. Detection of Oxidized PP2A by Carboxymethylation

Pre-OCs were treated with M-CSF (30 ng/mL) and LPS (50 ng/mL) in the presence or absence of DAC (7 μM) for 2 d, and further steps were followed according to methods previously described [10]. The lysate was subjected to immunoprecipitation with 1 µg of PP2A-specific Ab, and HRP-conjugated streptavidin was used to detect immunocomplexes labeled with N-(biotinoyl)-N’-(iodoacetyl) ethylenediamine (BIAM) (Invitrogen) for development with an enhanced chemiluminescence kit.

### 2.9. Transfection of siRNA

Small interfering RNA (siRNA) against PP2A (a pool of 3 target-specific 19–25 nt siRNAs designed to knock down gene expression) or scrambled siRNA (scRNA) was transfected into pre-OCs that had previously been incubated with M-CSF and RANKL for 40 h using Lipofectamine 3000 as described [9].

### 2.10. Statistical Analysis

All experiments were repeated at least three times. The data are expressed as the mean ± standard deviation. Statistical analysis was performed using Student’s *t*-test when two groups were compared or by one-way ANOVA followed by Bonferroni posttests when multiple groups were compared. Two-way ANOVA was used when two variables were analyzed. A *p* value less than 0.05 was considered statistically significant.

## 3. Results

### 3.1. Dauricine (DAC) Attenuates LPS-Induced Bone Loss in Mice

**Figure 1:**
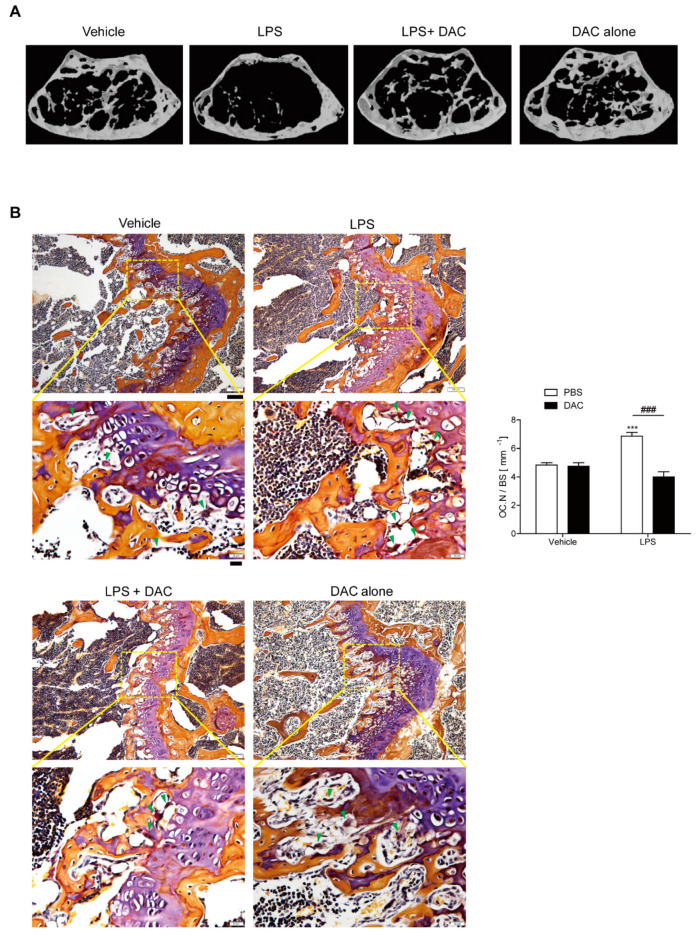
Dauricine (DAC) attenuates lipopolysaccharide (LPS)-induced bone loss in mice. (**A**) Representative μCT images of distal femora in a region 0.45 mm in length and located 0.55 mm below the growth plate to show the density of the trabecular bone that represents the outcome of active bone remodeling. Mice were treated with PBS (*n* = 5), LPS (5 mg/kg/week) (*n* = 5), LPS+DAC (2.5 mg/kg/d) (*n* = 5), or DAC only (2.5 mg/kg/d) (*n* = 5). Mice were treated for 3 weeks beginning at the age of 10 weeks. (**B**) To examine tartrate-resistant acid phosphatase (TRAP)-positive osteoclasts (OCs) in vivo, mouse femora were excised, cleaned with a soft tissue, and decalcified in EDTA. Representative histological sections of the distal femoral metaphyses of mice from each of the 4 groups were stained with TRAP to identify OCs (indicated by arrow-heads) to calculate OC.N/BS (OC number divided by total bone surface). Under the microscope, most OCs were found near to growth plates where active bone remodeling occurred, leading to the corresponding bone phenotype as an outcome. Scale bar: 100 μm in the representative photos. Inset shows a higher magnification of the image (Scale bar: 20 μm). *** *p* < 0.001, compared with PBS-injected mice. ### *p* < 0.001 compared with LPS-injected mice. Two-way ANOVA, followed by Bonferroni posttests, was used to compare the effect of DAC (*p* < 0.001), the effect of LPS (*p* < 0.05), and interactions (*p* < 0.001).

Femurs from mice treated with DAC or vehicle after the injection of LPS or PBS were analyzed using µCT in order to investigate the effect of DAC on LPS-induced inflammatory bone loss. A 2.5 mg/kg dose of DAC exhibited protection from bone loss compared to doses of 1.0 mg/kg and 5 mg/kg (Figure S1). Body weight was not significantly changed among the four groups at the age of 13 weeks. The analysis of µCT revealed significant bone loss with decreased bone mineral density (BMD), bone volume (BV/TV), and trabecular thickness (Tb. Th), and increased trabecular spaces (Tb. Sp.) in LPS-treated mice compared to in the PBS control (Figure 1B and Table 1). The circulating MCP-1 level was increased in response to LPS (Table 1).

By contrast, co-treatment with DAC prevented LPS-induced bone loss (Figure 1B, Table 1). The injection of DAC with LPS enhanced the BMD, BV/TV, and Tb. Th., and diminished the enlargement of Tb. Sp. more than LPS alone (Table 1), but DAC alone did not exhibit any significant differences compared with the vehicle control (Figure 1B, Table 1). As shown in Figure 1B, OC.N./BS (the ratio of the OC number to total bone surface area) as analyzed by in vivo TRAP staining was also significantly reduced by the injection of DAC into LPS-treated mice.

An in vivo bone resorption marker, CTX-1, which was enhanced by LPS treatment, was also decreased after treatment with DAC. However, no significant differences for serum ALP or osteocalcin, markers of bone formation in vivo, were observed for combined injections of DAC with LPS compared with LPS alone (Table 1). The same pattern was found for the number of osteoblasts OBs identified by H&E staining (Figure S2). The enhancement of serum MCP-1 by LPS was also attenuated by DAC (Table 1). Next, we determined whether DAC was associated with the attenuation of oxidative stress. LPS-injected mice exhibited elevated levels of serum ROS, whereas treatment with DAC significantly reduced ROS (Table 1).

### 3.2. DAC Decreases Differentiation and Activity of OCs upon LPS Stimulation In Vitro

**Figure 2:**
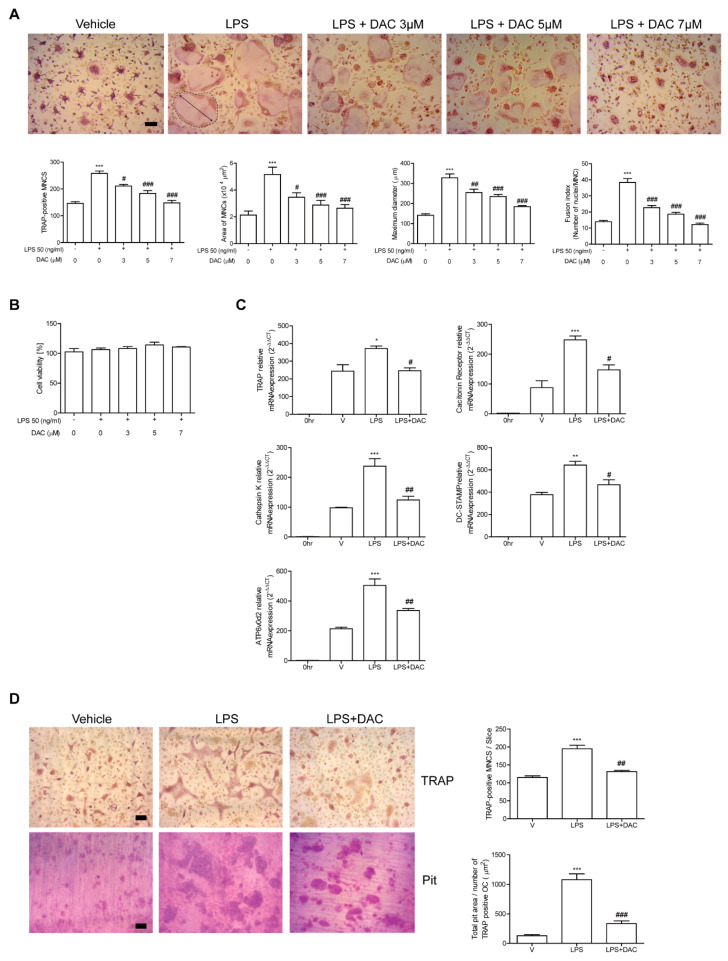
DAC decreases the differentiation and activity of OCs upon LPS stimulation in vitro. Bone marrow derived macrophages (BMMs) were cultured with M-CSF (30 ng/mL) and RANKL (40 ng/mL) for 40 h and further incubated with M-CSF and LPS (50 ng/mL) in the presence or absence of DAC (3 μM, 5 μM, or 7 μM) for 48 h (A, B) or 24 h (C). PBS was used as a vehicle. After fixation with formaldehyde, TRAP-positive multinucleated cells (MNCs) were counted. More than 70 cells were randomly selected for measuring the area (marked with dotted line), maximum diameter (double arrow of the formed OCs), and fusion index (the number of nuclei per TRAP-positive MNCs). Representative photos are shown. Scale bar: 100 μm in the representative OC photos (**A**). MTT assay was used to determine cell viability, and no significant difference was found compared with PBS-treated cells at these concentrations of DAC (**B**). RNA from cells stimulated with LPS in the presence or absence of DAC (7 μM) was analyzed by qPCR. The expression level before RANKL pre-treatment was set to be 1 (**C**). Mature OCs were further incubated on whole dentine slices with M-CSF and LPS in the presence or absence of DAC (7 μM) for 4 d. After TRAP staining, the cells were removed, and the slices were stained with toluidine blue. Representative photos of TRAP-positive OCs and resorption pits are shown. Scale bar: 100 μm in the representative photos. Total pit area/number of TRAP-positive OCs was calculated (**D**). * *p* < 0.05; ** *p* < 0.01; *** *p* < 0.001 compared with PBS-treated pre-OCs. # *p* < 0.05; ## *p* < 0.01; ### *p* < 0.001 compared with LPS-treated cells. Similar results were obtained from three independent experiments.

Since the participation of OCs in the protective effect of DAC on LPS-induced bone loss was indicated by our in vivo data, we assessed the effects of DAC on OCs upon LPS stimulation to investigate whether it applied to in vitro systems. The administration of LPS to RANKL-pretreated OC precursor cells induced the cells to differentiate into OCs. The OCs were fully activated after 48 h of LPS exposure as assessed by counting the TRAP-positive MNCs and measuring the mRNA expression of the OC-specific genes, TRAP, cathepsin K, DC-STAMP, ATP6v0d2, and calcitonin receptor. OC differentiation induced by LPS has been reported [11,12]. The number of TRAP-positive MNCs was reduced by DAC in a concentration-dependent manner (Figure 2A), although the viability of the cells was not affected by DAC under the assayed conditions (Figure 2B). Consistent with these results, DAC attenuated the mRNA expression of OC-specific genes including TRAP, calcitonin receptor, cathepsin K, DC-STAMP, and ATP6v0d2 (Figure 2C). The surface area and maximum diameter of the OCs and the fusion index that was calculated based on the number of nuclei per OC were also decreased by DAC treatment (Figure 2A).

Next, the effect of DAC on in vitro bone resorption activity of DAC after LPS stimulation was assessed using dentine slices. Generated mature OCs were incubated with or without DAC in the presence of LPS on the bone. The addition of DAC significantly reduced the total pit area/number of OCs in the LPS-treated cells (Figure 2D), implying that OC activity was affected by DAC.

### 3.3. DAC Decreases Both NF-kB Activation and the Up-Regulation of Cytosolic Reactive Oxygen Species (cROS) Induced by LPS Stimulation in OCs

**Figure 3:**
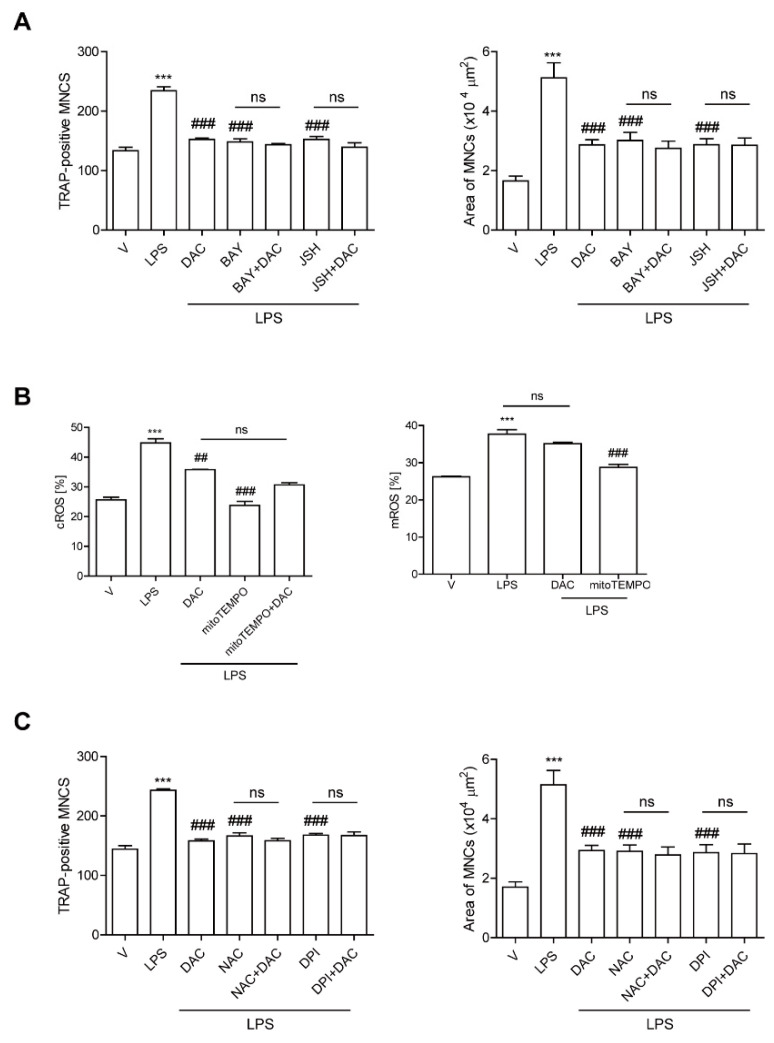
DAC decreases both NF-kB activation and the up-regulation of cytosolic reactive oxygen species (cROS) upon LPS stimulation in OCs. BMMs were prepared, incubated with RANKL (40 ng/mL) in the presence of M-CSF (30 ng/mL) for 40 h, and washed. Then, the cells were further incubated with BAY 11-7082 (3 μM) or JSH-23 (10 μM), which was chosen for 50% inhibition, and DPI (5 nM) (**A**) or NAC (3 mM) (**C**) and then stimulated with LPS (50 ng/mL) and M-CSF (30 ng/mL) in the presence or absence of DAC (7 μM) for 48 h. Cells were fixed after 48 h, and the number of TRAP-positive MNCs in each culture was counted. Cells were stimulated with LPS (50 ng/mL) and M-CSF (30 ng/mL) in the presence or absence of DAC (7 μM) for 24 h and 16 h to determine cytosolic ROS and mitochondrial ROS by flow cytometry using H^2^DCF-DA and mito-SOX red, respectively (**B**). *** *p* < 0.001 compared with RANKL-pretreated cells treated with vehicle (V). ## *p* < 0.01; ### *p* < 0.001 compared with LPS-treated cells. ns, no significant difference between 2 samples. Similar results were obtained in three independent experiments.

Since our previous data demonstrated that LPS-induced bone loss was attenuated by inhibiting NF-κB activation in OCs [9] and DAC has been reported to inhibit the NF-κB signaling pathway [19,20], we hypothesized that DAC may inhibit OC formation through attenuating NF-κB signaling in LPS-induced OCs. To investigate this, we assessed the effect of DAC on LPS-induced OC formation in the presence or absence of pharmacological inhibitors of NF-κB signaling pathways: agents for the blockade of nuclear translocation of p65 (JSH23) and that of IκBα phosphorylation (BAY 11-7082). While both inhibitors significantly decreased the TRAP-positive MNCs and the OC area induced by LPS, the effect of DAC was totally abolished when DAC was added to the cells along with each inhibitor (Figure 3A).

We also previously demonstrated that LPS induces OC differentiation by stimulating cROS [12] and mitochondrial ROS (mROS) production [10]. That prompted us to wonder whether DAC may decrease ROS to reduce LPS-induced OC formation. DAC dramatically decreased cROS at 24 h when the maximum level of cROS was induced by LPS. No changes in the mROS induced by LPS were observed in response to DAC treatment (Figure 3B). When a ROS scavenger, N-acetyl cysteine NAC, or an inhibitor of NADPH oxidase, diphenyleneiodonium chloride (DPI), was co-applied with DAC to LPS-induced OCs, the inhibitory effects of DAC on the number of OCs and the OC area were completely abolished (Figure 3C).

### 3.4. DAC Decreases LPS-Induced Oxidation of PP2A to Block NF-kB Activation via Decreasing the ROS Level in OCs

Since the inhibitory effect of DAC on OC formation was completely eliminated by both NF-κB inhibition and decreasing the ROS level, we suspected that both pathways were linked to the mechanism DAC uses to inhibit OC formation. Then, we wondered whether the decreased ROS due to DAC affected NF-κB signaling pathways by reducing the target molecules, thus blocking NF-κB activation. Since recent studies have reported that the inhibition of serine/threonine-protein phosphatase 2A (PP2A) results in the activation of the IKKα/IκBα/NF-κB pathway [26] and PP2A inactivation is mediated via LPS-induced ROS [27], we hypothesized that LPS-induced ROS may oxidize PP2A for inactivation, leading to NF-κB activation.

**Figure 4:**
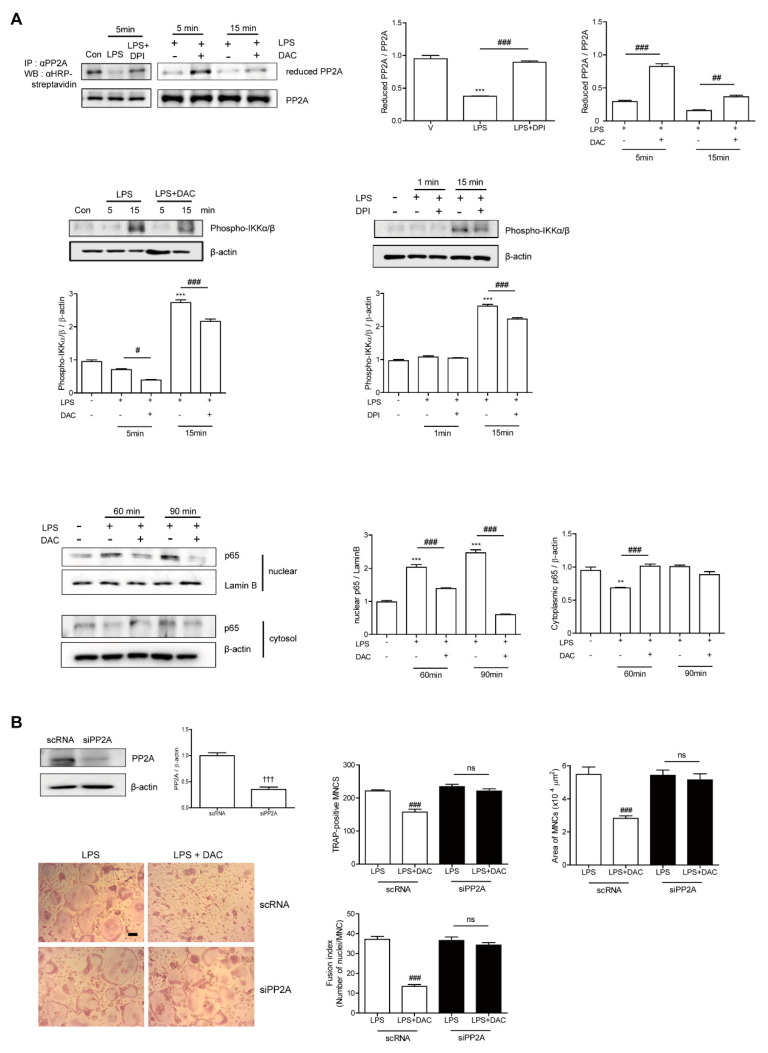
DAC decreases LPS-induced oxidation of PP2A to block NF-kB activation via decreasing the ROS level in OCs. Prepared BMMs were incubated with M-CSF and RANKL for 40 h, washed, and further incubation with M-CSF under indicated conditions (DPI, 5 nM; DAC, 7 μM) in the presence of LPS (50 ng/mL) was performed. After the labeling of the cell lysate using N-(biotinoyl)-N’-(iodoacetyl) ethylenediamine BIAM, immunoprecipitation (IP) was performed with anti-PP2A and followed by HRP–streptavidin immunoblotting to evaluate the reduced form of PP2A. Whole cell extracts were analyzed with an antibody against phospho-IKKα/β. After fractionation into cytoplasmic and nuclear parts, each was evaluated by Western blot analysis with antibodies against p65. The quantification of phospho-IKKα/β and p65, normalized to β-actin and lamin B1, respectively, was plotted (**A**). Cells were transfected with 50 nM of scRNA or siPP2A and further incubated for 48 h with LPS and M-CSF, and the number of TRAP-positive MNCs was counted. Reduced expression of PP2A by PP2A silencing was confirmed by western blot (WB). After fixation, the number of TRAP-positive MNCs, area, and fusion index of the OCs in each culture were analyzed (**B**). Uncropped WB images were shown in Figure S3. Dotted boxes indicated the cropped region for representative images in (**A**,**B**). ** *p* < 0.01; *** *p* < 0.001 compared with RANKL-pretreated cells treated with vehicle (V). # *p* < 0.05; ## *p* < 0.01; ### *p* < 0.001 compared with LPS-treated cells. ††† *p* < 0.001 compared with scRNA-treated cells. ns, no significant difference between 2 samples. Similar results were obtained in three independent experiments.

Next, we assessed whether DAC reduced the oxidized form of PP2A to attenuate NF-κB signaling. As shown in Figure 4A, LPS decreased the level of reduced PP2A, leading to increased phospho-IKKα/β, whereas DAC treatment regained the reduced form of PP2A and decreased phospho-IKKα/β in the presence of LPS. A similar phenomenon was found with DPI treatment. The enhanced nuclear localization of p65 after LPS exposure was attenuated by DAC. To confirm the role of PP2A in the effect of DAC on OCs, we silenced PP2A. When PP2A was silenced, the inhibitory effect of DAC on the OC number, OC area, and fusion index was abolished completely (Figure 4B), whereas the knock-down of PP2A showed a tendency to increase TRAP-positive MNCs and the fusion index of the OCs compared to the scRNA-treated control upon LPS stimulation, although the changes were not significant (Figure 4B).

## 4. Discussion

We have demonstrated that DAC, an isoquinoline alkaloid derived from R. *menispermi,* exhibits anti-inflammatory activity [18,19]. It protected mice against the inflammatory bone loss induced by LPS. The administration of LPS in vivo induced a marked decrease in bone density with an increased number of OCs without any change in serum levels of ALP and osteocalcin, in vivo bone formation markers, suggesting that OCs were responsible for the LPS-induced bone loss. Increased bone resorption was not followed by up-regulated bone formation in LPS-induced bone loss, suggesting an uncoupling of bone remodeling, although a coupled bone remodeling has been shown in ovariectomy -induced [24,28] or cholesterol-induced bone loss [29]. Uncoupled bone remodeling has been reported in myeloma-related bone disease [30], and progranulin-induced bone loss in female mice [31]. In addition, uncoupling has been proposed under prolonged inflammation as a mechanism of periodontal bone loss [32]. After the administration of DAC to LPS-injected mice, the serum levels of MCP-1 and ROS were significantly reduced, suggesting that DAC attenuated the systemic inflammation and oxidative stress that accompanied the inflammatory bone loss induced by LPS. In vitro, DAC decreased the OC number, OC area, and OC-specific genes, and bone resorption on dentine slices was also reduced by DAC, indicating that DAC inhibited not only OC differentiation but also OC activity. Although DAC decreased RANKL-induced OC formation in vitro (Figure S4), DAC alone did not induce any significant differences in bone density compared to the vehicle control. It could be due to the much lower RANKL level under in vivo physiological conditions than that under in vitro assay conditions. The up-regulated expression of RANKL has been reported under pathological conditions such as loss of ovarian function [33].

DAC with LPS exhibited maximum protection at a dose of 2.5 mg/kg without any significant change in OBs. However, it is plausible that DAC did not confer any protection from bone loss at a higher dose due to reduced bone formation. DAC has been demonstrated to be therapeutically effective in rodents at 5–10 mg/kg for inflammatory processes induced by ischemia/reperfusion [18], 5–20 mg/kg to protect from cecal ligation and puncture-induced acute lung injury [19], and 6–12 mg/kg to suppress pancreatic tumor growth [34]. In vitro inhibition of NF-kB has been reported to be within the ranges of 1–10 μM and 5–40 μM in macrophages [19] and colon cancer cells [20], respectively. The plasma level of DAC has been reported to be 16 μg/mL after 15 min of intravenous injection (15 mg/kg) in the rat [35]. Assuming a similar pattern was applied, the injected DAC dose of 2.5 mg/kg could induce a plasma level of 2.7 μg/mL, within the range of our used concentrations in vitro. Since DAC has been applied to healthy male volunteers at the dose of 60–180 mg [36], it is possible that the protective dose, 2.5 mg/kg (equivalent to 150 mg/60 kg), of DAC could be used for translational application.

To investigate the mechanism by which DAC affected OCs, the effect of DAC on LPS-induced signaling was examined in RANKL-treated pre-OCs. LPS has been shown to activate NF-κB signaling [9], thereby promoting osteoclastogenesis. The importance of NF-κB signaling in bone has been demonstrated by NF-κB1 and NF-κB2 double KO mice exhibiting low bone density caused by impaired osteoclastogenesis [15], although the detailed mechanism behind this phenotype has not been clearly elucidated. Two pharmacological inhibitors of NF-κB signaling, BAY 11-7082 and JSH23, abolished the inhibitory effect of DAC on OC formation induced by LPS, suggesting that the inhibitory effect of DAC on OC formation was mainly due to the blockade of NF-κB signaling.

DAC also reduced the ROS that were up-regulated by LPS. The removal of ROS by NAC and DPI totally diminished the inhibition by DAC of TRAP-positive MNCs and the OC area, indicating that the effect of DAC was mediated by ROS. The blockade of ROS synthesis by DPI, an inhibitor of NADPH oxidase, reduced the oxidized form of PP2A induced by LPS, indicating that ROS acted upstream of NF-κB signaling. The decreasing effect of DAC on the number and area of OCs was completely abolished by PP2A silencing, indicating that PP2A is necessary for the effects of DAC on OCs.

Collectively, our data demonstrated that DAC acted as an inhibitor to decrease the differentiation and activity of OCs through the ROS/PP2A/NF-κB axis. In agreement with our findings, several studies have also showed an association of ROS with NF-κB signaling [27,37,38,39,40,41,42], although the detailed mechanisms by which ROS affects NF-κB signaling have not been clearly elucidated yet. NF-κB activation by ROS could be highly cell-type specific, with distinct mechanisms [37]. The direct addition of ROS to Jurkat T cells has been reported to activate NF-κB [38]. In T cells, H_2_O_2_ induces the Syk-mediated Y42 phosphorylation of IκBα or the IKK-induced S32 and 36 phosphorylation of IκBα, depending on the expression of the inositol phosphatase SHIP-1 [39,40]. In epithelial cells, H_2_O_2_ triggers IKK complex activation through PKD activation [41]. Pervanadate induces IκBα tyrosine phosphorylation in all studied cell-types in a c-Src-dependent fashion [42]. ROS-mediated SHP2 and PP2A inactivation leads to NF-κB activation in human endothelial cells [27].

Our present study showed that LPS induced ROS and subsequently oxidized PP2A to inactivate its enzymatic activity, leading to IKKα/β phosphorylation and, later, the nuclear localization of p65, suggesting that the effect of LPS on NF-κB activation in OCs was modulated by the ROS/PP2A/IKKα/β/p65 axis. DAC reduced oxidized PP2A and p65 nuclear translocation via decreasing the ROS levels that were increased by LPS.

## 5. Conclusions

Taken together, these results suggest that DAC attenuated inflammatory bone loss by decreasing ROS, which targeted PP2A, subsequently leading to decreased NF-κB activation in OCs, suggesting that PP2A could be a potential therapeutic target for inflammatory bone loss.

## Figures and Tables

**Table 1:** Trabecular microarchitecture and biochemical markers of LPS with or without dauricine treatment in mice.

Parameter	Vehicle (PBS)		LPS	
	PBS	Dauricine	PBS	Dauricine
BMD [mg/cm^3^]	198.8 ± 4.726	190.6 ± 4.279	142.9 ± 5.044 ^a″^	174.6 ± 5.397 ^b″^
BV/TV [%]	15.42 ± 0.469	14.96 ± 0.357	12.23 ± 0.297 ^a″^	14.24 ± 0.484 ^b′^
Tb.Th [μm]	67.47 ± 2.146	65.22 ± 1.773	55.03 ± 1.421 ^a″^	62.04 ± 2.493 ^b^
Tb.Sp [μm]	346.9 ± 4.308	361.5 ± 7.363	448.1 ± 9.309 ^a″^	352.7 ± 12.1 ^b″^
ALP [U/L]	46.56 ± 2.015	43.78 ± 1.330	44.20 ± 1.379	45.06 ± 3.048
OCN [ng/mL]	25.73 ± 1.285	26.11 ± 1.381	28.28 ± 1.786	28.96 ± 1.220
CTX-1 [ng/mL]	26.31 ± 0.343	25.88 ± 1.126	52.10 ± 4.405 ^a″^	23.76 ± 2.190 ^b″^
MCP-1 [pg/mL]	182.8 ± 11.88	186.7 ± 16.39	272.0 ± 26.36 ^a^	175.5 ± 29.50 ^b^
H_2_O_2_ [μM]	46.08 ± 1.065	47.71 ± 0.736	50.52 ± 0.408 ^a″^	47.84 ± 0.531 ^b″^

PBS (*n* = 5); LPS (dissolved in PBS, 5 mg/kg) (*n* = 5); LPS+dauricine (dissolved in PBS, 2.5 mg/kg) (*n* = 5); dauricine only (*n* = 5). Data are represented as means ± SDs. Differences between groups were analyzed by two-way ANOVA, followed by Bonferroni posttests to compare the effect of lycorine (BMD; *p* < 0.05, Tb.Sp and CTX-1; *p* < 0.001), the effect of LPS (Tb.Th and H_2_O_2_; *p* < 0.01, BMD, Tb.Sp, bone volume (BV/TV), CTX-1; *p* < 0.001) and interaction (Tb.Th and MCP-1; *p* < 0.05, BV/TV and H2O2; *p* < 0.01, BMD, Tb.Sp and CTX-1; *p* < 0.001). ^a^ *p* < 0.05, ^a^^″^ *p* < 0.001 compared with vehicle-injected mice. ^b^ *p* < 0.05, ^b^^′^ *p* < 0.01, ^b^^″^ *p* < 0.001 compared with LPS-injected mice.

## Supplementary Materials

The following are available online at https://www.mdpi.com/2076-3921/9/7/588/s1, Figure S1: DAC attenuates LPS-induced bone loss in mice, Figure S2: DAC does not show any significant change in LPS-treated mice, Figure S3: WB images corresponding to Figure 4, Figure S4: DAC decreases differentiation of OCs upon RANKL stimulation in vitro.
