# Supplementary material for: Dauricine Protects from LPS-Induced Bone Loss via the ROS/PP2A/NF-κB Axis in Osteoclasts
Source: Antioxidants (Basel). 2020 Jul 6;9(7):588. doi: 10.3390/antiox9070588 (PMC7402093; doi:10.3390/antiox9070588)
Supplement: Supplementary file 1 [file antioxidants-09-00588-s001.pdf]

Supplementary Figure 1. DAC attenuates LPS-induced bone loss in mice.

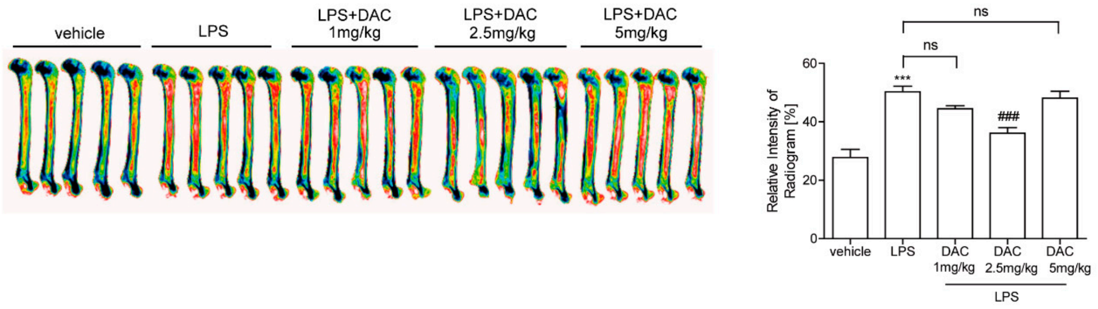

Representative images and relative intensities of the distal femurs from X-ray radiograms mice treated with PBS (n=5), LPS (5 mg/kg/week) (n = 5), or LPS+DAC (1.0 mg/kg/d; n=5, 2.5 mg/kg/d; n=5, 5 mg/kg/d; n=5) were measured using the Image J program. Bone density: blue>green>yellow>red>white. *** $p<0.001$ compared with PBS-treated mice. ### $p<0.001$ compared with LPS-treated mice. ns, no significant difference between 2 samples. Similar results were obtained in three independent experiments.

Supplementary Figure 2. DAC does not show any significant change in LPS-treated mice.

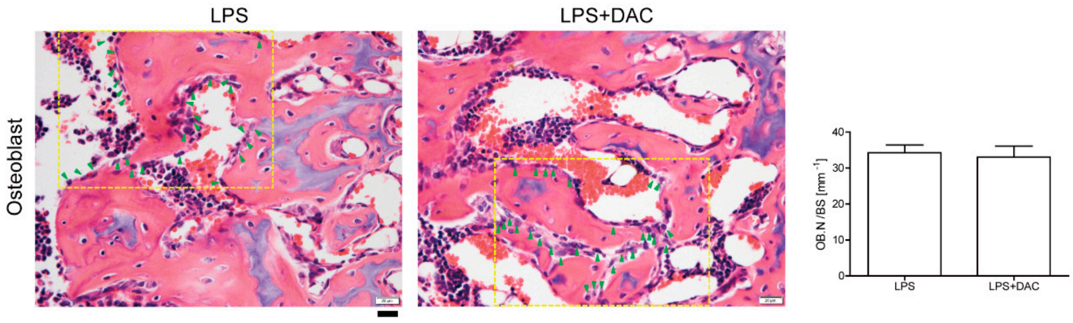

After H&E staining, OBs were identified to be "plump cuboidal cells with perinuclear clear zone on osteoid surface" [1]. Representative histologic sections of distal femoral metaphysis of each group were stained for H&E to identify OBs (green arrows in boxed area) (original magnification x400) with OB.N/BS (OB number over total bone surface). Scale bar: 20 μm in representative photos.

Supplementary Figure 3. WB images corresponding to Fig. 4.

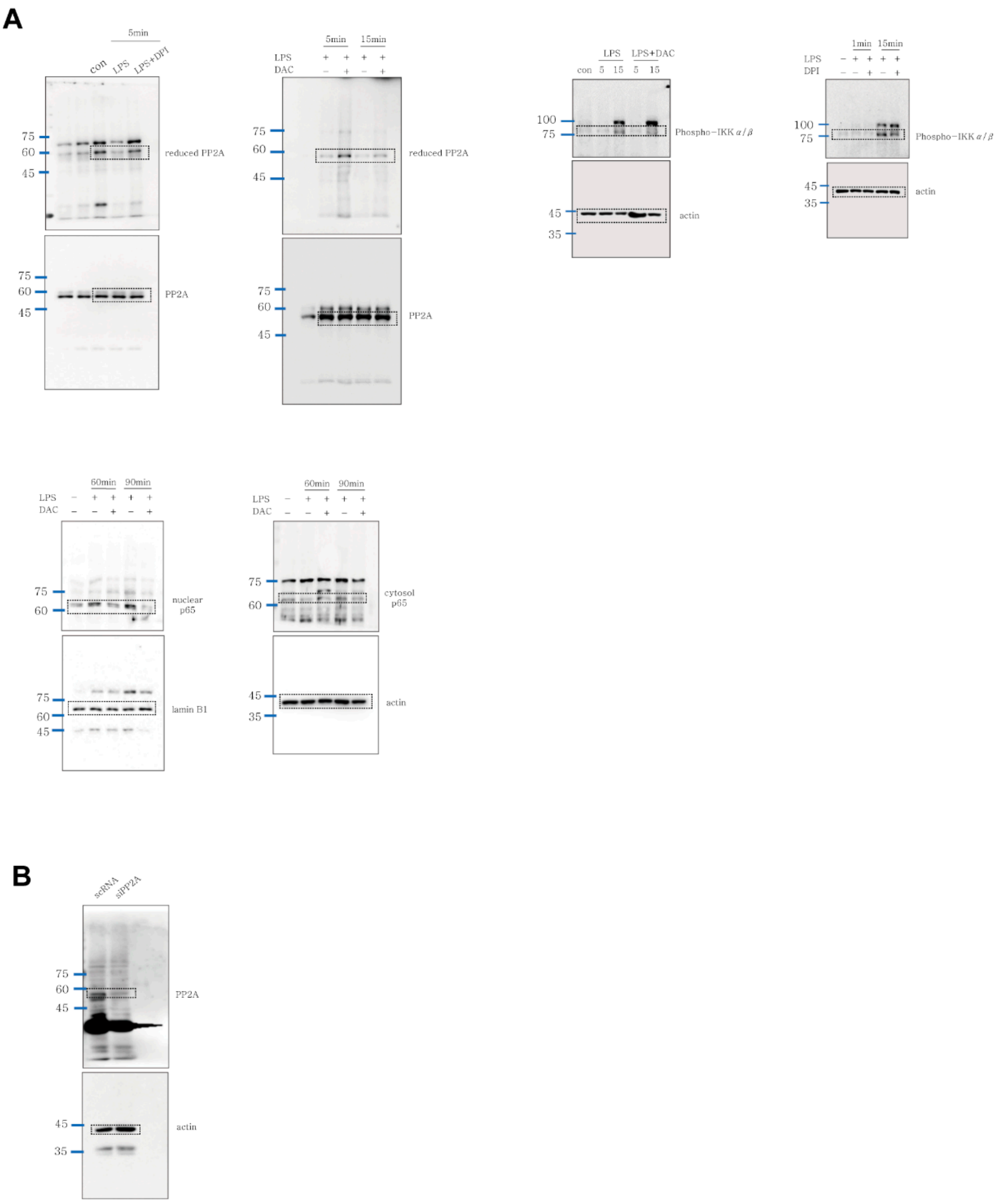

Supplementary Figure 4. DAC decreases differentiation of OCs upon RANKL stimulation *in vitro*.

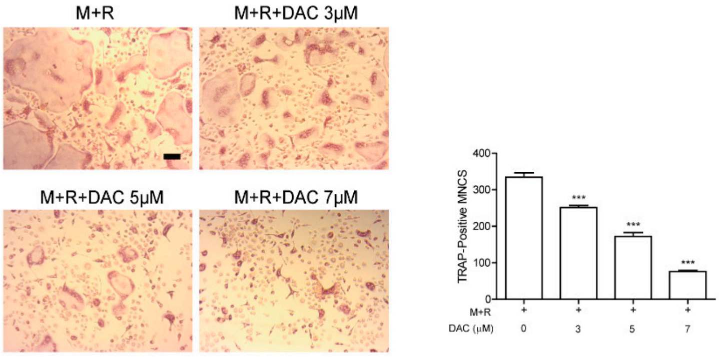

BMMs (10^4 cells/well) were prepared and incubated with RANKL (40 ng/ml) and M-CSF (30 ng/ml) in the absence or presence of DAC (3 μM, 5 μM, 7 μM) for 72 h. Cells were fixed to count TRAP-positive MNCs. *** $p < 0.001$ compared with PBS-treated group.

[1] Jilka, RL.; Weinstein, RS.; Bellido, T.; Roberson, P.; Parfitt, AM.; et al. Increased bone formation by prevention of osteoblast apoptosis with parathyroid hormone. *J Clin Invest*. **1999**, *104*, 439-446.
